# Deep learning algorithm for predicting left ventricular systolic dysfunction in atrial fibrillation with rapid ventricular response

**DOI:** 10.1093/ehjdh/ztae062

**Published:** 2024-08-19

**Authors:** Joo Hee Jeong, Sora Kang, Hak Seung Lee, Min Sung Lee, Jeong Min Son, Joon-myung Kwon, Hyoung Seok Lee, Yun Young Choi, So Ree Kim, Dong-Hyuk Cho, Yun Gi Kim, Mi-Na Kim, Jaemin Shim, Seong-Mi Park, Young-Hoon Kim, Jong-Il Choi

**Affiliations:** Division of Cardiology, Department of Internal Medicine, Korea University College of Medicine, Korea University Anam Hospital, 73 Goryeodae-ro, Seongbuk-gu, Seoul 02841, Republic of Korea; Medical Research Team, Medical AI Co., Seoul, Republic of Korea; Medical Research Team, Medical AI Co., Seoul, Republic of Korea; Medical Research Team, Medical AI Co., Seoul, Republic of Korea; Medical Research Team, Medical AI Co., Seoul, Republic of Korea; Medical Research Team, Medical AI Co., Seoul, Republic of Korea; Division of Cardiology, Department of Internal Medicine, Korea University College of Medicine, Korea University Anam Hospital, 73 Goryeodae-ro, Seongbuk-gu, Seoul 02841, Republic of Korea; Division of Cardiology, Department of Internal Medicine, Korea University College of Medicine, Korea University Anam Hospital, 73 Goryeodae-ro, Seongbuk-gu, Seoul 02841, Republic of Korea; Division of Cardiology, Department of Internal Medicine, Korea University College of Medicine, Korea University Anam Hospital, 73 Goryeodae-ro, Seongbuk-gu, Seoul 02841, Republic of Korea; Division of Cardiology, Department of Internal Medicine, Korea University College of Medicine, Korea University Anam Hospital, 73 Goryeodae-ro, Seongbuk-gu, Seoul 02841, Republic of Korea; Division of Cardiology, Department of Internal Medicine, Korea University College of Medicine, Korea University Anam Hospital, 73 Goryeodae-ro, Seongbuk-gu, Seoul 02841, Republic of Korea; Division of Cardiology, Department of Internal Medicine, Korea University College of Medicine, Korea University Anam Hospital, 73 Goryeodae-ro, Seongbuk-gu, Seoul 02841, Republic of Korea; Division of Cardiology, Department of Internal Medicine, Korea University College of Medicine, Korea University Anam Hospital, 73 Goryeodae-ro, Seongbuk-gu, Seoul 02841, Republic of Korea; Division of Cardiology, Department of Internal Medicine, Korea University College of Medicine, Korea University Anam Hospital, 73 Goryeodae-ro, Seongbuk-gu, Seoul 02841, Republic of Korea; Division of Cardiology, Department of Internal Medicine, Korea University College of Medicine, Korea University Anam Hospital, 73 Goryeodae-ro, Seongbuk-gu, Seoul 02841, Republic of Korea; Division of Cardiology, Department of Internal Medicine, Korea University College of Medicine, Korea University Anam Hospital, 73 Goryeodae-ro, Seongbuk-gu, Seoul 02841, Republic of Korea

**Keywords:** Artificial intelligence, Deep learning, Left ventricular ejection fraction, Atrial fibrillation, Rate control

## Abstract

**Aims:**

Although evaluation of left ventricular ejection fraction (LVEF) is crucial for deciding the rate control strategy in patients with atrial fibrillation (AF), real-time assessment of LVEF is limited in outpatient settings. We aimed to investigate the performance of artificial intelligence–based algorithms in predicting LV systolic dysfunction (LVSD) in patients with AF and rapid ventricular response (RVR).

**Methods and results:**

This study is an external validation of a pre-existing deep learning algorithm based on residual neural network architecture. Data were obtained from a prospective cohort of AF with RVR at a single centre between 2018 and 2023. Primary outcome was the detection of LVSD, defined as a LVEF ≤ 40%, assessed using 12-lead electrocardiography (ECG). Secondary outcome involved predicting LVSD using 1-lead ECG (Lead I). Among 423 patients, 241 with available echocardiography data within 2 months were evaluated, of whom 54 (22.4%) were confirmed to have LVSD. Deep learning algorithm demonstrated fair performance in predicting LVSD [area under the curve (AUC) 0.78]. Negative predictive value for excluding LVSD was 0.88. Deep learning algorithm resulted competent performance in predicting LVSD compared with N-terminal prohormone of brain natriuretic peptide (AUC 0.78 vs. 0.70, *P* = 0.12). Predictive performance of the deep learning algorithm was lower in Lead I (AUC 0.68); however, negative predictive value remained consistent (0.88).

**Conclusion:**

Deep learning algorithm demonstrated competent performance in predicting LVSD in patients with AF and RVR. In outpatient setting, use of artificial intelligence–based algorithm may facilitate prediction of LVSD and earlier choice of drug, enabling better symptom control in AF patients with RVR.

## Graphical Abstract

**Graphical Abstract:**
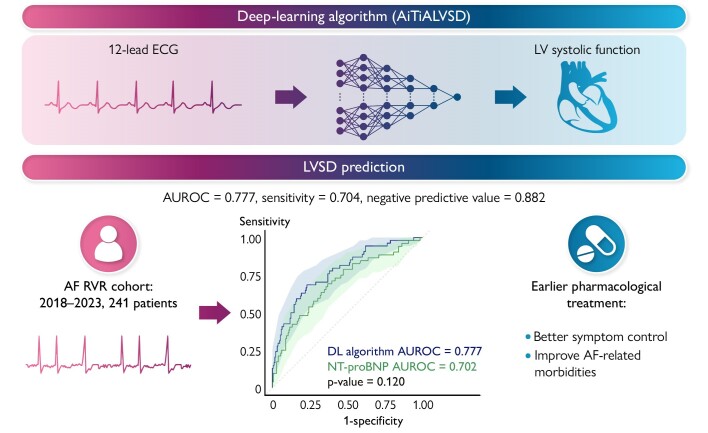
ECG, electrocardiography; LV, left ventricular; AF, atrial fibrillation; RVR, rapid ventricular response; LVSD; left ventricular systolic dysfunction; AUROC, area under the receiver operating characteristic curve.

## Introduction

Atrial fibrillation (AF) is the most prevalent sustained cardiac arrhythmia in adults, exerting a significant burden on patients and healthcare system.^1^ Initial detection of AF prompts a comprehensive cardiovascular assessment for affected individuals.^2^ In the case of AF, echocardiographic evaluation is important for evaluating underlying substrate and guiding subsequent treatment decisions. The assessment of left ventricular (LV) systolic function is mandatory within the standard diagnostic evaluation package for AF.^1^

Atrial fibrillation with rapid ventricular response (RVR) contributes significantly to patient-related symptoms, healthcare utilization, and the development of heart failure.^3^ Effective rate control is an integral part of management in patients with AF and RVR, leading to improved AF-related symptoms and a reduction in heart failure hospitalizations.^4^ Accordingly, current guideline recommends that prompt evaluation of LV function should precede rate control and long-term rhythm control.^1,5^ However, conducting real-time assessments of LV function through transthoracic echocardiography in an outpatient, emergency, or primary care setting poses considerable challenges. Over the past two decades, there has been a substantial increase in the volume of transthoracic echocardiography in clinical practice.^6^ Moreover, the recent COVID-19 pandemic has aggravated the supply–demand mismatch for routine outpatient echocardiography, resulting in waiting times exceeding 6 weeks.^7^

The use of 12-lead electrocardiography (ECG) is recommended for all patients with AF, providing a simple and readily accessible tool in the outpatient or primary care setting.^1^ Predictive algorithms using deep learning techniques have demonstrated remarkable performance in various medical applications, particularly in image detection and clinical outcome prediction.^8–10^ Various ECG-based deep learning algorithms have been introduced to predict LV systolic function.^11,12^ To date, there was little evidence to utilize ECG-based deep learning algorithm in patients with AF, especially those in RVR. Thus, this study aimed to assess the efficacy of a deep learning algorithm in predicting LV systolic dysfunction (LVSD) among patients with AF and RVR and to investigate its feasibility in clinical practice.

## Methods

### Database

This is a validation study of a pre-existing deep learning algorithm that predicts LVSD from 12-lead ECG.^12^ External data were obtained from the established observational cohort of the AF RVR registry at the Korea University, Anam Hospital. The AF RVR registry was initially established to investigate the clinical outcomes of patients with AF experiencing RVR. Adult patients were prospectively enrolled in the registry between October 2018 and May 2023, if they met the study criteria of (i) being diagnosed with AF and (ii) having available 12-lead ECG records of AF or atrial flutter with a ventricular rate ≥ 100 b.p.m. Twelve-lead ECGs that were successfully converted to an analysable format (XML file) via central viewer server (MUSE, GE Healthcare) were included in the analysis. Exclusions from the analysis encompassed patients who (i) lacked a 12-lead ECG record for AF and RVR or had ECGs that were not converted to analysable format, (ii) were not examined using transthoracic echocardiography, or (iii) withdrew their informed consent. Additionally, patients who did not undergo echocardiography within 2 months of 12-lead ECG were excluded. The registry protocols adhered to the principles of the Declaration of Helsinki. Written informed consent was waived by the Institutional Review Board of the Korea University Medicine, Anam Hospital, due to the retrospective nature of this study, and previous informed consent was obtained upon enrolment to the registry.

### Outcome measurement and definition of variables

The primary outcome was the detection of LVSD, defined as a LV ejection fraction (LVEF) ≤ 40%, using data from the 12-lead ECG. The secondary outcome involved predicting LVSD using the 1-lead (Lead I) of the 12-lead ECG. The 12-lead ECG at the day of the registry enrolment was used for the analysis. If there were multiple ECGs at the same day (of enrolment), the initial ECG presented with AF and RVR was used. During transthoracic echocardiography, the LVEF was measured using the modified Simpson’s method. In patients with AF during echocardiography, LVEF was calculated from the average value of three consecutive beats.^13^ Only formal echocardiographic records were included in the analysis. N-terminal prohormone of brain natriuretic peptide (NT-proBNP) measured at the time of AF with RVR or the nearest value was collected.

### Development of the deep learning algorithm

In 2019, Kwon *et al*.^12^ developed a model utilizing artificial intelligence for screening LVSD through the analysis of 12-lead ECG. In 2023, following additional clinical research and development, this algorithm-based software, known as AiTiALVSD version 1.00.00, obtained approval and clearance from the Ministry of Food and Drug Safety of the Republic of Korea, recognized as an artificial intelligence/machine learning–based Software as a Medical Device (SaMD).^14–17^ The AiTiALVSD artificial intelligence algorithm was developed using a residual neural network architecture and exclusively relies on digital signals with a frequency of 500 Hz from 12-lead raw ECG data as its input, without incorporating additional clinical variables. The output is an LVSD probability score, presented as a decimal ranging from 0 to 100, precise to the first decimal place. A defined probability score of 9.7 serves as the threshold distinguishing high and low risk, a parameter established to yield a performance level approaching 90% sensitivity. For Lead I analysis, the software’s artificial intelligence algorithm was adapted to use Lead I from a 12-lead ECG as input, maintaining the same output configuration as previously described. Further details about the development of deep learning algorithm are specified in the Supplementary material. The AiTiALVSD is not opened to the public as the algorithm is proprietary to the company (Medical AI Co., Ltd.).

### Statistical analysis

The diagnostic efficacy of AiTiALVSD was assessed utilizing various metrics, including the area under the receiver operating characteristic curve (AUROC), area under the precision-recall curve (AUPRC), sensitivity, specificity, positive predictive value, and negative predictive value (NPV). Each metric was reported with a 95% confidence interval (CI) for primary and secondary outcomes. The continuous LVSD probability score was employed for calculating AUROC and AUPRC, while other metrics utilized a predetermined cut-off of 9.7. N-terminal proBNP was used to predict LVSD, and the cut-off value to predict LVSD was calculated based on Youden index. Data underwent statistical analysis using appropriate tests, including the Student’s *t*-test, Mann–Whitney *U* test, *χ*^2^ test, and Fisher’s exact test. Multivariable logistic regression analysis was used to identify predictors of the primary outcome. Statistical significance was ascertained at values of *P* < 0.05. Comprehensive analyses were conducted employing R software version 4.1.0 and Python 3.8.

## Results

**Figure 1:**
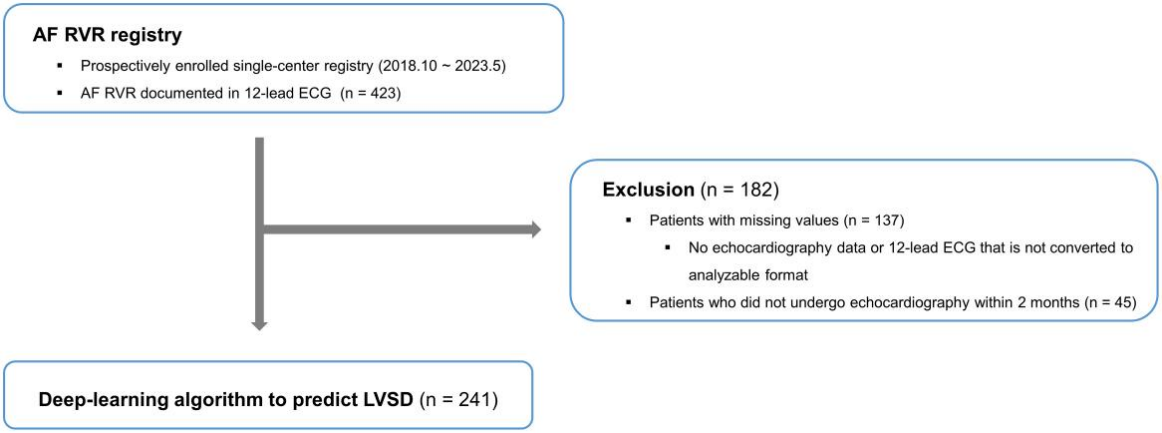
Flowchart of the patient selection process. AF, atrial fibrillation; RVR, rapid ventricular response; ECG, electrocardiography; LVSD, left ventricular systolic dysfunction.

A total of 423 patients with AF and RVR were enrolled in the registry between October 2018 and May 2023 (*Figure 1*). Exclusions included patients (i) with missing echocardiography data or 12-lead ECG not converted to an analysable format (*n* = 137) and (ii) those who did not undergo echocardiography within 2 months (*n* = 45), resulting in the inclusion of 241 consecutive patients for analysis. The mean age of the patients was 65.4 ± 12.0 years, and 160 (66.4%) of them were male (*Table 1*). The baseline heart rate during RVR was 120.6 ± 15.6 b.p.m.. Ninety-two patients (38.2%) had paroxysmal AF, and 10 presented with atrial flutter (4.1%). In total, 183 (75.9%) of the 12-lead ECGs were performed in outpatient clinics, and 42 (38.2%) were performed in the emergency department. The mean duration from 12-lead ECG to echocardiography was 17.5 ± 15.9 days, and 181 (75.1%) of the patients had AF or atrial flutter during the echocardiographic assessment. The mean LVEF was 47.9 ± 10.8%, and the mean left atrial diameter was 44.2 ± 6.5 mm. Transthoracic echocardiography confirmed LVSD in 54 patients (22.4%). Tachycardia-induced cardiomyopathy or AF were the most common aetiologies of LVSD (74.1%; Supplementary material online, *Table S1*). Patients with LVSD more frequently maintained AF (or atrial flutter) during the echocardiographic assessment and exhibited significantly increased left atrial diameter and NT-proBNP.

**Table 1:** Baseline characteristics of the study patients

	Total cohort	LVSD	No LVSD	*P*-value
	(*n* = 241)	(*n* = 54)	(*n* = 187)	
Age (years)	65.4 ± 12.0	63.8 ± 13.1	65.8 ± 11.7	0.272
Male sex	160 (66.4)	43 (79.6)	117 (62.6)	0.243
Body mass index (kg/m^2^)	25.2 ± 3.7	25.4 ± 5.1	25.2 ± 3.3	0.752
Heart rate (b.p.m.)	120.6 ± 15.6	123.0 ± 16.8	120.0 ± 15.2	0.212
Place during AF RVR				0.906
Outpatient clinic	183 (75.9)	37 (68.5)	146 (78.1)	
Admission	16 (6.6)	5 (9.3)	11 (5.9)	
Emergency department	42 (17.4)	12 (22.2)	30 (16.0)	
Paroxysmal AF	92 (38.2)	18 (33.3)	74 (39.6)	0.952
Atrial flutter	10 (4.1)	2 (3.7)	8 (4.3)	0.853
Duration of AF (years)	2.4 ± 3.9	2.3 ± 3.7	2.4 ± 4.0	0.872
Echocardiography				
Time to echocardiography (days)	17.5 ± 15.9	16.8 ± 15.8	17.7 ± 15.9	0.719
Rhythm during echocardiography				0.008
Sinus rhythm	60 (24.9)	3 (5.6)	57 (30.5)	
AF or atrial flutter	181 (75.1)	51 (94.4)	130 (69.5)	
Left ventricular diastolic dimension (mm)	47.6 ± 6.3	51.4 ± 8.5	46.5 ± 5.1	<0.001
Left ventricular systolic dimension (mm)	32.8 ± 7.5	40.3 ± 9.5	30.6 ± 5.1	<0.001
Septal thickness (mm)	10.2 ± 1.7	10.4 ± 1.7	10.2 ± 1.7	0.314
Posterior wall thickness (mm)	9.6 ± 1.4	9.7 ± 1.1	9.5 ± 1.5	0.529
Aorta thickness (mm)	32.4 ± 4.4	32.0 ± 5.5	32.7 ± 4.0	0.418
Left atrial diameter (mm)	44.2 ± 6.5	46.5 ± 6.5	43.6 ± 6.3	0.004
Left atrial volume index (kg/m^2^)	48.6 ± 19.4	51.9 ± 19.8	47.9 ± 19.4	0.494
Left ventricular mass (g)	168.7 ± 52.1	193.8 ± 70.0	161.5 ± 43.3	0.002
Left ventricular mass index (g/m^2^)	95.2 ± 24.7	106.7 ± 31.2	91.9 ± 21.4	0.002
LVEF (%)	48.0 ± 10.8	31.4 ± 7.1	52.8 ± 5.8	<0.001
E	73.7 ± 21.5	75.7 ± 17.7	73.2 ± 22.4	0.506
*e*′	7.3 ± 2.3	6.5 ± 1.9	7.5 ± 2.3	0.011
*E*/*e*′	11.3 ± 5.8	13.1 ± 6.2	10.8 ± 5.6	0.023
Tricuspid regurgitation velocity (m/s)	2.5 ± 0.4	2.3 ± 0.2	2.5 ± 0.4	0.020
Estimated pulmonary artery pressure (mmHg)	34.8 ± 7.8	34.6 ± 8.4	34.8 ± 7.7	0.874
CHA_2_DS_2_-VASc score	2.9 ± 1.5	2.8 ± 1.4	2.9 ± 1.6	0.789
NT-proBNP (pg/mL)	1493.3 ± 2235.1	2796.8 ± 3642.2	1120.8 ± 1437.1	<0.001
Comorbidities				
Hypertension	122 (50.6)	30 (55.6)	92 (49.2)	0.954
Diabetes mellitus	44 (18.3)	9 (16.7)	35 (18.7)	0.998
Dyslipidaemia	84 (34.9)	14 (25.9)	70 (37.4)	0.641
Thyroid disease	36 (14.9)	10 (18.5)	26 (13.9)	0.951
Vascular disease	18 (7.5)	6 (11.1)	12 (6.4)	0.855
Heart failure	118 (49.0)	53 (98.2)	65 (34.8)	<0.001
Ischaemic stroke	29 (12.0)	3 (5.6)	26 (13.9)	0.599
Electrocardiography				
PR interval (ms)	155.0 ± 32.0	151.7 ± 8.6	156.4 ± 38.7	0.843
QRS duration (ms)	91.6 ± 17.8	96.7 ± 19.5	90.2 ± 17.0	0.017
QT interval (ms)	324.6 ± 32.8	327.7 ± 37.1	323.7 ± 31.5	0.424
QTc interval (ms)	456.7 ± 41.8	464.6 ± 48.9	454.4 ± 39.4	0.117
*P*-axis	105.7 ± 93.4	157.5 ± 110.3	85.0 ± 82.9	0.201
*R*-axis	44.3 ± 54.3	53.0 ± 63.8	41.7 ± 51.2	0.180
*T*-axis	32.2 ± 57.9	47.4 ± 69.1	27.8 ± 53.7	0.028
AiTiALVSD-12L score	18.1 ± 23.7	37.9 ± 31.9	12.3 ± 16.9	<0.001
AiTiALVSD-1L score	19.9 ± 18.4	30.3 ± 22.8	17.0 ± 15.8	<0.001

LVSD, left ventricular systolic dysfunction; AF, atrial fibrillation; b.p.m., beats per minute; RVR, rapid ventricular response; LVEF, left ventricular ejection fraction; NT-proBNP, N-terminal prohormone of brain natriuretic peptide.

### Model performance

**Figure 2:**
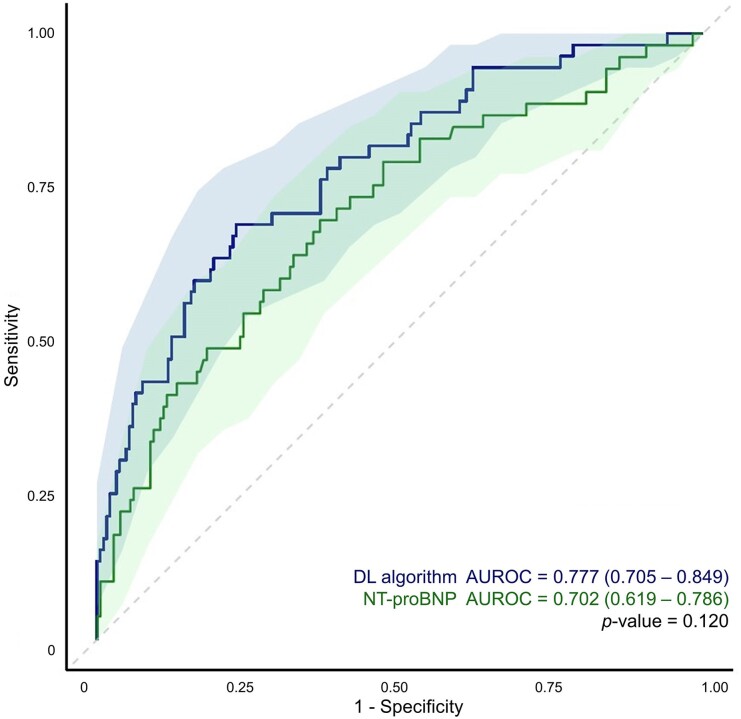
Receiver operating characteristic curve of the primary outcome. The receiver operating characteristic curve (line) is presented with its 95% confidence interval (shades). DL, deep learning; NT-proBNP, N-terminal prohormone of brain natriuretic peptide; AUROC, area under the receiver operating characteristic curve.

The deep learning algorithm, using 12-lead ECG, demonstrated an AUROC of 0.78 (95% CI 0.71–0.85) for predicting LVSD (*Table 2* and *Figure 2*). The NPV for excluding LVSD was 0.88 (95% CI 0.83–0.94). N-terminal proBNP was also utilized to predict LVSD, which resulted a cut-off value of 911.2 pg/mL. The deep learning algorithm revealed competent performance compared with NT-proBNP in predicting LVSD in patients with AF and RVR.

**Table 2:** Model performance for evaluating primary outcome

	Deep learning algorithm	NT-proBNP
AUROC (95% CI)	0.777 (0.705–0.849)	0.702 (0.619–0.786)
AUPRC (95% CI)	0.321 (0.252–0.388)	0.449 (0.340–0.564)
Sensitivity (95% CI)	0.704 (0.582–0.825)	0.692 (0.567–0.818)
Specificity (95% CI)	0.642 (0.573–0.710)	0.632 (0.562–0.702)
Positive predictive value (95% CI)	0.362 (0.270–0.454)	0.350 (0.257–0.442)
Negative predictive value (95% CI)	0.882 (0.828–0.937)	0.878 (0.822–0.934)

AUROC, area under the receiver operating characteristic curve; CI, confidence interval; AUPRC, area under the precision-recall curve; NT-proBNP, N-terminal prohormone of brain natriuretic peptide.

The predictive performance of the deep learning algorithm using Lead I was lower than that of the 12-lead ECG–based prediction (AUROC 0.68; 95% CI 0.59–0.77; *Table 3*; Supplementary material online, *Figure S1*). However, the NPV remained consistent at 0.88 (95% CI 0.82–0.95).

**Table 3:** Model performance in secondary outcome

	Deep learning algorithm
AUROC (95% CI)	0.678 (0.591–0.765)
AUPRC (95% CI)	0.432 (0.340–0.564)
Sensitivity (95% CI)	0.796 (0.689–0.904)
Specificity (95% CI)	0.444 (0.373–0.515)
Positive predictive value (95% CI)	0.293 (0.219–0.366)
Negative predictive value (95% CI)	0.883 (0.818–0.948)

AUROC, area under the receiver operating characteristic curve; CI, confidence interval; AUPRC, area under the precision-recall curve.

Patients who underwent echocardiography within 2 weeks (*n* = 144) were further evaluated for the model performance in predicting LVSD (see Supplementary material online, *Table S2* and *Figure S2*). In this subgroup, the predictive performance of the deep learning algorithm showed improvement for both 12-lead ECG–based algorithm (AUROC 0.80, 95% CI 0.71–0.90; AUPRC 0.65, 95% CI 0.51–0.77) and the Lead I–based algorithm (AUROC 0.75, 95% CI 0.66–0.85; AUPRC 0.48, 95% CI 0.34–0.63). Additionally, the NPV was enhanced, reaching its peak in the Lead I–based algorithm [0.96 (95% CI 0.91–1.00)].

**Figure 3:**
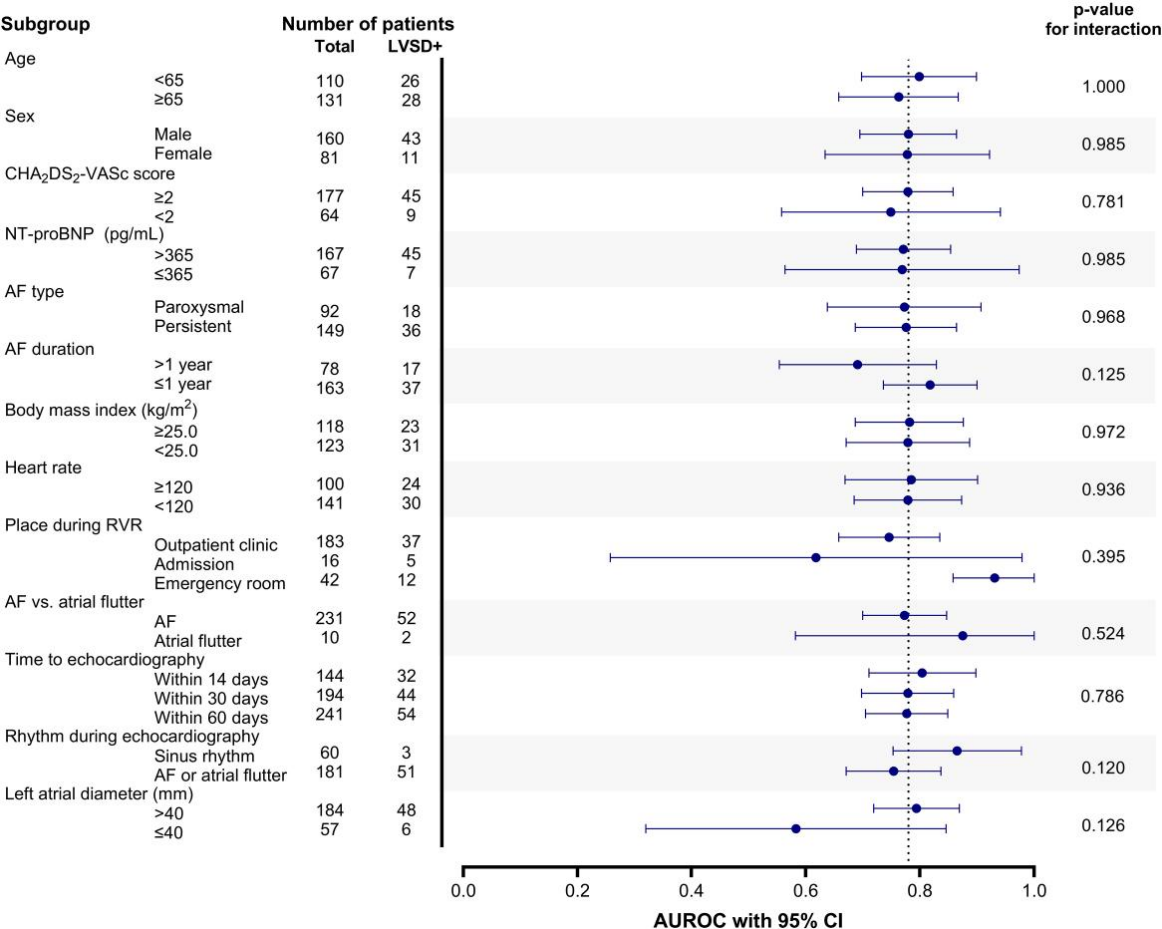
Subgroup analysis. Area under the receiver operating characteristic curve of deep learning algorithm to predict left ventricular systolic dysfunction (0.777) was set as reference. LVSD, left ventricular systolic dysfunction; AUROC, area under the receiver operating characteristic curve; CI, confidence interval; NT-proBNP, N-terminal prohormone of brain natriuretic peptide; AF, atrial fibrillation; RVR, rapid ventricular response.

The model’s performance for the primary outcome was assessed across various subgroups (see Supplementary material online, *Table S3*; *Figure 3*). Although no significant interaction was observed between different subgroups, there was a tendency towards better performance in certain scenarios, including (i) patients who underwent echocardiography within 14 days (AUROC 0.80, 95% CI 0.71–0.90), (ii) AF detected within 1 year (AUROC 0.82, 95% CI 0.74–0.90), (iii) AF with RVR occurring in the emergency department (AUROC 0.93, 95% CI 0.86–1.00), (iv) patients with atrial flutter during RVR (AUROC 0.88, 95% CI 0.58–1.00), and (v) patients who converted to sinus rhythm during echocardiography assessment (AUROC 0.87, 95% CI 0.75–0.98).

Four variables were identified as predictors of the primary outcome (*Table 4*). An increase in QRS duration, NT-proBNP, or LV mass and a decrease in LV mass index were found to be associated with an increased risk of having LVSD.

**Table 4:** Predictors of primary outcome

	Odds ratio	95% confidence interval	*P*-value
Place during AF RVR	0.932	0.543–1.568	0.789
Atrial flutter	2.340	0.333–16.281	0.381
Heart rate (b.p.m.)	1.010	0.942–1.073	0.784
QRS duration (ms)	1.030	1.010–1.051	0.017
QT interval (ms)	1.010	0.951–1.051	0.725
QTc interval (ms)	1.000	0.961–1.041	0.875
*R*-axis	0.990	0.990–1.000	0.093
*T*-axis	1.000	0.990–1.000	0.306
Female sex	1.786	0.427–7.463	0.427
Age at AF diagnosis	1.010	0.951–1.073	0.763
Persistent AF	1.310	0.527–3.320	0.558
NT-proBNP	1.000	1.000–1.000	0.005
New onset AF	1.246	0.449–3.525	0.676
Duration of AF (years)	1.062	0.942–1.197	0.348
Time to echocardiography (days)	0.990	0.961–1.010	0.290
AF or atrial flutter during echocardiography	1.030	0.346–3.096	0.957
Body surface area (m^2^)	0.000	0.000–0.990	0.053
Left ventricular diastolic dimension (mm)	1.073	0.852–1.433	0.579
Left ventricular systolic dimension (mm)	1.105	1.000–1.234	0.050
Septal thickness (mm)	1.492	0.869–2.801	0.162
Posterior wall thickness (mm)	1.020	0.595–1.859	0.938
Aorta thickness (mm)	0.970	0.896–1.051	0.517
Left atrial diameter (mm)	1.000	0.932–1.073	0.990
Left ventricular mass (g)	1.083	1.000–1.174	0.051
Left ventricular mass index (g/m^2^)	0.844	0.719–0.980	0.033
Estimated pulmonary artery pressure (mmHg)	1.000	0.942–1.051	0.898
CHA_2_DS_2_-VASc score	0.691	0.320–1.448	0.330
Body mass index (kg/m^2^)	1.020	0.869–1.197	0.832
Dyslipidaemia	1.094	0.507–2.340	0.819
Thyroid disease	2.181	0.827–6.050	0.120
Hypertension	1.616	0.595–4.482	0.346
Diabetes mellitus	1.185	0.333–4.263	0.789
Vascular disease	4.055	0.819–23.336	0.096
Heart failure	2.460	0.852–7.463	0.101
Ischaemic stroke	3.254	0.566–19.886	0.193

Increase of odds ratio means increased probability of having left ventricular systolic dysfunction.

AF, atrial fibrillation; RVR, rapid ventricular response; NT-proBNP, N-terminal prohormone of brain natriuretic peptide.

## Discussion

The present study investigated the efficacy of deep learning algorithms in predicting LVSD and its applicability in the clinical practice for diagnosing patients with AF and RVR. Despite the inherent challenge in predicting LV function in patients presenting with AF and RVR, the 12-lead ECG–based algorithm demonstrated fair performance (AUROC 0.78). Patients with a shorter time window between 12-lead ECG and echocardiography exhibited improved performance (AUROC 0.80). Deep learning algorithms yielded excellent NPVs in both the 12-lead and 1-lead ECG–based algorithm. The high NPV in patients with AF and RVR potentially facilitates informed choices in drug selection for rate and rhythm control in an outpatient setting.

### Prediction of heart failure with reduced left ventricular ejection fraction

The deep learning algorithm employed in this study has undergone validation in large-sized cohort in previous studies.^12,17^ The 12-lead ECG–based algorithm, initially developed with data from 22 765 patients, was externally validated (*n* = 5901), demonstrating good performance in predicting heart failure with reduced LVEF (AUROC 0.84).^12^ Additionally, the developmental data included ECGs with AF or atrial flutter, constituting less than 20% of the total cohort. Regardless of algorithm types, heart rate and the presence of AF or atrial flutter were identified as important variables influencing predictive performance. In essence, predicting LV function was more challenging in patients presenting with tachycardia or AF. The sensitivity of LVSD prediction using 12-lead ECG was 70.4%, whereas it was improved as 78.1% in subgroup that underwent echocardiography within 14 days. Similarly, in single-lead ECG–based prediction, the sensitivity was 79.6%, which was improved as 93.8% in 14 days interval subgroup. Based on the cohort of patients with AF and RVR, this study successfully validated the performance of deep learning algorithm in predicting LVSD.

### Clinical implications

Patients detected with AF are recommended for early evaluation, focusing on assessing structural heart disease and substrate. Particularly, patients in the acute phase of AF with RVR require prompt evaluation of the LV function to guide further treatment strategies. Distinguishing LVSD in patients with AF is essential, as it limits the spectrum of drug choices for rate rhythm control. Particularly, calcium channel blockers or class Ic antiarrhythmic drugs are contraindicated in this population. However, patients with stable AF are typically evaluated and treated in outpatient settings, where real-time, quantitative assessment of LVEF is challenging. In addition, presence of AF and RVR may impair the diagnostic value of laboratory markers such as NT-proBNP.^18^ Consequently, deep learning algorithms predicting LVEF may provide additional information for further decision-making. In our study, the validation of deep learning algorithms in the AF RVR cohort revealed fair AUROC and high NPV that was higher than that of NT-proBNP. The high NPV is advantageous in excluding individuals with LVSD, allowing for the informed selection of appropriate drugs for rate or rhythm control. Therefore, the deep learning–based prediction of LVSD may extend clinical information needed for further treatment in circumstances where echocardiography is not feasible. Although deep learning algorithm may not fully substitute echocardiography, earlier prediction of LV function using a deep learning algorithm may be beneficial, leading to earlier pharmacotherapy to relieve AF-related morbidities. For instance, deep learning algorithm could be used to exclude patients with heart failure with reduced EF, and appropriate rate control and antiarrhythmic drugs could be prescribed without several weeks of delay. Earlier pharmacotherapy enabled by deep learning algorithm is consistent to the paradigm of early rhythm control in AF, which may reduce cardiovascular events and further progression of AF.^19,20^ High NPV observed in single-lead ECG–based prediction may expand its applicability into various settings such as wearable ECGs (smartwatch) and in-hospital telemetry monitoring.

### Assessment of left ventricular ejection fraction during atrial fibrillation? Or sinus rhythm?

In our cohort, although all patients presented AF (or atrial flutter) and RVR at enrolment, 24.9% restored sinus rhythm when the echocardiography was performed. Assessing echocardiography during AF may underestimate the true LVEF, especially in those who present RVR. In addition, there is a high chance of LVEF improvement in patients who restored sinus rhythm.^21^ There is a gap of evidence in the guidelines, which do not specify the treatment strategies according to the rhythm that the LVEF was assessed.^1,5^ On the other hand, the LVSD predicted by deep learning algorithm is not influenced by the cardiac rhythm—whether the rhythm is AF or sinus. Subgroup that restored sinus rhythm during echocardiography showed higher predictive performance compared with that maintained AF or atrial flutter (AUC 0.865 vs. 0.754) without statistical difference (*P* = 0.120, *Figure 3*; Supplementary material online, *Table S2*). This may indicate that deep learning algorithm may have higher predictive value in detecting LVSD due to structural substrate (such as dilated cardiomyopathy or ischaemic heart disease) than those with LVSD related to AF. In addition, applying deep learning algorithm to predict LVSD may overcome the practical limitation—of whether the patient rhythm is AF or not—that disturbs identification of the ‘true’ LVEF.

### Limitations

This study has several limitations. First, the lack of strict protocols in terms of the time interval between 12-lead ECG and echocardiography introduces variability among patients. Echocardiographic evaluation during AF may underestimate the true LVEF, and restoration of sinus rhythm in the meanwhile may improve LVEF, leading to the discrepancy of predicted LVEF (by deep learning algorithm) and calculated LVEF (by echocardiography). This variability reflects the real-world outpatient setting, where the time until echocardiographic assessment can be even longer than the predefined 6 weeks. Second, the inclusion of only 12-lead ECGs converted into an analysable format in the central server may introduce bias. Since ECGs that were taken by portable device required additional step of sending the ECGs to the central server, excluded cases may represent a significant proportion taken in acute or critically ill condition (i.e. in the intensive care unit), potentially limiting the applicability of deep learning–based prediction in patients with acute and haemodynamically unstable conditions. Third, although our study focused on identifying the presence of LVSD, the aetiology of LVSD was not considered in the prediction. Left ventricular systolic dysfunction in AF is not equivalent to LVSD in sinus rhythm, since AF itself may contribute to the development of LVSD. In a previous study, three key features in beat-to-beat patterns of AF were relevant to AF-induced heart failure, which resulted in high specificity (91.4%) and positive predictive value (87.0%).^22^ Deep learning algorithm used in our study was originally developed in mostly sinus rhythms, and beat-to-beat variability (RR intervals) was not initially considered in the model development. However, a small proportion of AF or atrial flutter (11.8%) were included in original model, and beat-to-beat variability may had been trained as a hidden feature to predict LVSD in AF. Further consideration of the aetiology of LVSD such as AF-related LVSD that is reversible with sinus conversion may also facilitate clinical intervention. Fourth, intrinsic limitations may lie in the prediction model and model performance when applying it in clinical practice. For instance, NPV of 0.88 may lead to inappropriate prescription of contraindicated medication in up to 12% of patients with AF and RVR. Whether the benefit of earlier pharmacotherapy to be free from RVR and restore sinus rhythm outweighs the hazards of inappropriate drug choice is unknown. Treatment strategy based on the deep learning algorithm should be accompanied by physician’s clinical insight and relevant clinical circumstances. Lastly, the study investigated the performance of the deep learning algorithm based on echocardiographic assessment of LVEF, and a direct comparison with conventional echocardiography was not conducted. Further prospective trials are needed to assess the non-inferiority of the deep learning algorithm compared with conventional echocardiography and warrant its broader use in clinical practice.

## Conclusion

In patients presenting with AF and RVR, the 12-lead ECG–based deep learning algorithm demonstrated good performance in predicting LVSD. The high NPV, particularly in excluding LVSD, potentially facilitates rapid and enhanced selection of drug therapy in an outpatient, emergency or primary care setting. Early assessment of LV systolic function using the deep learning algorithm may prove beneficial for the guideline-directed rate control strategy in patients with AF and RVR, leading to better symptom control and optimization of AF-related morbidities.

## Supplementary Material

ztae062_Supplementary_Data

## Data Availability

The data underlying this article are available in the article and in its supplementary material.

## References

[1] Hindricks G, Potpara T, Dagres N, Arbelo E, Bax JJ, Blomstrom-Lundqvist C, et al 2020 ESC guidelines for the diagnosis and management of atrial fibrillation developed in collaboration with the European Association for Cardio-Thoracic Surgery (EACTS): the task force for the diagnosis and management of atrial fibrillation of the European Society of Cardiology (ESC) developed with the special contribution of the European Heart Rhythm Association (EHRA) of the ESC. Eur Heart J 2021;42:373–498. 32860505 10.1093/eurheartj/ehaa612

[2] January CT, Wann LS, Calkins H, Chen LY, Cigarroa JE, Cleveland JC, et al 2019 AHA/ACC/HRS focused update of the 2014 AHA/ACC/HRS guideline for the management of patients with atrial fibrillation. A report of the American College of Cardiology/American Heart Association Task Force on Clinical Practice Guidelines and the Heart Rhythm Society. J Am Coll Cardiol 2019;74:104–132. 30703431 10.1016/j.jacc.2019.01.011

[3] Daoud EG, Weiss R, Bahu M, Knight BP, Bogun F, Goyal R, et al Effect of an irregular ventricular rhythm on cardiac output. Am J Cardiol 1996;78:1433–1436. 8970422 10.1016/s0002-9149(97)89297-1

[4] Van Gelder IC, Groenveld HF, Crijns HJ, Tuininga YS, Tijssen JG, Alings AM, et al Lenient versus strict rate control in patients with atrial fibrillation. N Engl J Med 2010;362:1363–1373. 20231232 10.1056/NEJMoa1001337

[5] Joglar JA, Chung MK, Armbruster AL, Benjamin EJ, Chyou JY, Cronin EM, et al 2023 ACC/AHA/ACCP/HRS guideline for the diagnosis and management of atrial fibrillation: a report of the American College of Cardiology/American Heart Association Joint Committee on Clinical Practice Guidelines. Circulation 2024;149:e1–e156. 38033089 10.1161/CIR.0000000000001193 PMC11095842

[6] Rahimi AR, York M, Gheewala N, Markson L, Hauser TH, Manning WJ. Trends in outpatient transthoracic echocardiography: impact of appropriateness criteria publication. Am J Med 2011;124:740–746. 21787903 10.1016/j.amjmed.2011.03.030

[7] Ng SM, Naqvi D, Bingcang J, Cruz G, Nose R, Lloyd G, et al Feasibility, diagnostic performance and clinical value of an abbreviated echocardiography protocol in an out-patient cardiovascular setting: a pilot study. Echo Res Pract 2022;9:8. 36104742 10.1186/s44156-022-00009-2 PMC9473732

[8] D'Ascenzo F, De Filippo O, Gallone G, Mittone G, Deriu MA, Iannaccone M, et al Machine learning-based prediction of adverse events following an acute coronary syndrome (PRAISE): a modelling study of pooled datasets. Lancet 2021;397:199–207. 33453782 10.1016/S0140-6736(20)32519-8

[9] Lu L, Zhu TT, Ribeiro AH, Clifton L, Zhao E, Zhou JD, et al Decoding 2.3 million ECGs: interpretable deep learning for advancing cardiovascular diagnosis and mortality risk stratification. Eur Heart J Digit Health 2024;5:247–259. 38774384 10.1093/ehjdh/ztae014 PMC11104458

[10] Kwon JM, Kim KH, Eisen HJ, Cho Y, Jeon KH, Lee SY, et al Artificial intelligence assessment for early detection of heart failure with preserved ejection fraction based on electrocardiographic features. Eur Heart J Digit Health 2021;2:106–116. 36711179 10.1093/ehjdh/ztaa015 PMC9707919

[11] Yagi R, Goto S, Katsumata Y, MacRae CA, Deo RC. Importance of external validation and subgroup analysis of artificial intelligence in the detection of low ejection fraction from electrocardiograms. Eur Heart J Digit Health 2022;3:654–657. 36710903 10.1093/ehjdh/ztac065 PMC9779862

[12] Kwon JM, Kim KH, Jeon KH, Kim HM, Kim MJ, Lim SM, et al Development and validation of deep-learning algorithm for electrocardiography-based heart failure identification. Korean Circ J 2019;49:629–639. 31074221 10.4070/kcj.2018.0446 PMC6597456

[13] Lang RM, Badano LP, Mor-Avi V, Afilalo J, Armstrong A, Ernande L, et al Recommendations for cardiac chamber quantification by echocardiography in adults: an update from the American Society of Echocardiography and the European Association of Cardiovascular Imaging. Eur Heart J Cardiovasc Imaging 2015;16:233–270. 25712077 10.1093/ehjci/jev014

[14] Kwon JM, Jo YY, Lee SY, Kang S, Lim SY, Lee MS, et al Artificial intelligence-enhanced smartwatch ECG for heart failure-reduced ejection fraction detection by generating 12-lead ECG. Diagnostics (Basel) 2022;12:654. 35328207 10.3390/diagnostics12030654 PMC8947562

[15] Lee BT, Jo YY, Lim SY, Song Y, Kwon JM. Efficient data augmentation policy for electrocardiograms. In: Proceedings of the 31st ACM International Conference on Information & Knowledge Management, Atlanta, GA, 2022. p.4153–4157. Association for Computing Machinery, New York, NY, USA.

[16] Lee Y, Choi B, Lee MS, Jin U, Yoon S, Jo Y-Y, et al An artificial intelligence electrocardiogram analysis for detecting cardiomyopathy in the peripartum period. Int J Cardiol 2022;352:72–77. 35122911 10.1016/j.ijcard.2022.01.064

[17] Jung YM, Kang S, Son JM, Lee HS, Han GI, Yoo AH, et al Electrocardiogram-based deep learning model to screen peripartum cardiomyopathy. Am J Obstet Gynecol MFM 2023;5:101184. 37863197 10.1016/j.ajogmf.2023.101184

[18] Richards M, Di Somma S, Mueller C, Nowak R, Peacock WF, Ponikowski P, et al Atrial fibrillation impairs the diagnostic performance of cardiac natriuretic peptides in dyspneic patients: results from the BACH study (Biomarkers in ACute Heart Failure). JACC Heart Fail 2013;1:192–199. 24621869 10.1016/j.jchf.2013.02.004

[19] Nattel S, Guasch E, Savelieva I, Cosio FG, Valverde I, Halperin JL, et al Early management of atrial fibrillation to prevent cardiovascular complications. Eur Heart J 2014;35:1448–1456. 24536084 10.1093/eurheartj/ehu028

[20] Kim D, Yang PS, You SC, Sung JH, Jang E, Yu HT, et al Treatment timing and the effects of rhythm control strategy in patients with atrial fibrillation: nationwide cohort study. BMJ 2021;373:n991. 33975876 10.1136/bmj.n991 PMC8111568

[21] Muller-Edenborn B, Minners J, Allgeier J, Burkhardt T, Lehrmann H, Ruile P, et al Rapid improvement in left ventricular function after sinus rhythm restoration in patients with idiopathic cardiomyopathy and atrial fibrillation. Europace 2019;21:871–878. 31157388 10.1093/europace/euz013

[22] Luongo G, Rees F, Nairn D, Rivolta MW, Dossel O, Sassi R, et al Machine learning using a single-lead ECG to identify patients with atrial fibrillation-induced heart failure. Front Cardiovasc Med 2022;9:812719. 35295255 10.3389/fcvm.2022.812719 PMC8918925

