# Supplementary material for: Deep learning algorithm for predicting left ventricular systolic dysfunction in atrial fibrillation with rapid ventricular response
Source: Eur Heart J Digit Health. 2024 Aug 19;5(6):683–91. doi: 10.1093/ehjdh/ztae062 (PMC11570393; doi:10.1093/ehjdh/ztae062)
Supplement: ztae062_Supplementary_Data [file ztae062_supplementary_data.docx]

**Supplementary Materials**

**Supplementary methods**

**#1. Architecture and transparency of AiTiALVSD (version 1.00.00)**

We developed a Residual Neural Network (ResNet) model for ECG classification utilizing PyTorch and Python. The architecture consists of a stem block, four residual blocks, and a fully connected network, with each block comprising a one-dimensional convolutional neural network, batch normalization, ReLU activations, and dropout. ECG data were obtained from four South Korean hospitals, using digitalized 12-lead ECGs recorded at a sampling rate of 500 Hz for a duration of 10 seconds. The dataset includes 364,845 records, divided into 291,564 training entries (79.9% of the total), 55,320 validation entries (15.1%), and 17,961 testing entries (5.0%). The original article analyzing this model's transparency is currently under peer review. The composition of the development data is presented in the table below.

|  | **Train**  **dataset** | | **Validation**  **dataset** | | **Test**  **dataset** | | **Total** | |
| --- | --- | --- | --- | --- | --- | --- | --- | --- |
| **Number of ECG record** | 291,564 | | 55,320 | | 17,961 | | 364,845 | |
| **Hospital** | | | | | | | |  |
| **Hospital A** | 193,702 | | 37,074 | | 11,883 | | 242,659 |  |
| **Hospital B** | 60,622 | | 11,353 | | 3,737 | | 75,712 |  |
| **Hospital C** | 22,053 | | 4,019 | | 1,378 | | 27,450 |  |
| **Hospital D** | 15,187 | | 2,874 | | 963 | | 19,024 |  |
| **Gender** | | | | | | | | |
| **Female** | 124,277 | 23,333 | | 7,688 | | 155,298 | | |
| **Male** | 162,139 | 30,945 | | 9,954 | | 203,038 | | |
| **Unknown** | 5,148 | 1,042 | | 319 | | 6,509 | | |
| **Age** | | | | | | | | |
| **~20** | 1,752 | 308 | | 101 | | 2,161 | | |
| **20~30** | 7,828 | 1,393 | | 555 | | 9,776 | | |
| **30~40** | 14,944 | 2,854 | | 1,002 | | 18,800 | | |
| **40~50** | 32,415 | 6,167 | | 1,937 | | 40,519 | | |
| **50~60** | 57,633 | 10,552 | | 3,934 | | 72,119 | | |
| **60~70** | 66,647 | 12,491 | | 3,926 | | 83,064 | | |
| **70~80** | 69,352 | 13,345 | | 4,047 | | 86,744 | | |
| **80~90** | 31,864 | 6,351 | | 1,968 | | 40,183 | | |
| **90~100** | 3,045 | 636 | | 132 | | 3,813 | | |
| **100~** | 45 | 5 | | 0 | | 50 | | |
| **Unknown** | 6,039 | 1,218 | | 359 | | 7,616 | | |
| **ECG Statement** | | | | | | | | |
| **Sinus rhythm** | 242,251 | 45,771 | | 14,805 | | 302,827 | | |
| **AF^a^** | 34,129 | 6,752 | | 2,215 | | 43,096 | | |
| **Others^b^** | 15,184 | 2,797 | | 941 | | 18,922 | | |

^a^ECGs that include the statements "atrial fibrillation," "afib," "atrial flutter/fibrillation," "a-flutter," or "atrial flutter,"

^b^ECGs classified as other rhythms not in AF or sinus rhythm.

Sinus rhythm, atrial fibrillation or flutter, and other rhythms constituted 11.7%, 12.2%, and 12.3% of the development, validation, and test sets, respectively. The ECGs were formatted as 12 × 5000 matrices and subsequently downsampled to enhance computational efficiency. Techniques such as data augmentation and Mixup methods were utilized to enhance model generalization. Ejection fraction (EF) values below 40% were labeled as positive, while EF values of 40% or higher were categorized as zero, in accordance with the European Society of Cardiology guidelines. During training, the model weights were updated using the Adam optimizer combined with a cosine warm-up optimization scheduler and a focal loss function. A population-based training scheduler optimized various hyperparameters over 150 epochs, and the optimal model architecture was determined based on the AUROC, AUPRC, loss, and F1-score performances on the validation set.

**#2. AiTiALVSD-1L: AI/ML algorithm to detect LVSD using lead I ECG**

In this investigation, a 1-Lead (lead I) AI/ML algorithm was utilized, which was derived from AiTiALVSD version 1.00.00. The modified algorithm retains the architectural framework of the original model; however, it has been specifically tailored to accept solely 1-Lead (lead I) electrocardiograms as input data.

**Supplementary Table 1. Etiology of LVSD**

|  | **LVSD (n=54)** |
| --- | --- |
| Tachycardia induced cardiomyopathy or AF-related | 40 (74.1) |
| Dilated cardiomyopathy | 6 (11.1) |
| Ischemic cardiomyopathy | 2 (3.7) |
| Hypertrophic cardiomyopathy | 1 (1.9) |
| Valvular heart disease | 1 (1.9) |
| Pacing induced cardiomyopathy | 1 (1.9) |
| Stress induced cardiomyopathy | 1 (1.9) |
| Unidentified | 2 (3.7) |

LVSD, left ventricular systolic dysfunction; AF, atrial fibrillation

**Supplemental Table 2. Model performance within the 14-day interval**

|  | **AUROC (95% CI)** | **AUPRC (95% CI)** | **Sensitivity (95% CI)** | **Specificity (95% CI)** | **Positive predictive value (95% CI)** | **Negative predictive**  **value (95% CI)** |
| --- | --- | --- | --- | --- | --- | --- |
| **AiTiALVSD (12-lead)** | 0.804 (0.711–0.898) | 0.646 (0.512–0.768) | 0.781 (0.638–0.924) | 0.625 (0.535–0.715) | 0.373 (0.257–0.489) | 0.909 (0.845–0.973) |
| **AiTiALVSD (1-lead)** | 0.751 (0.655–0.847) | 0.475 (0.342–0.628) | 0.938 (0.854–1.00) | 0.446 (0.354–0.538) | 0.326 (0.230–0.422) | 0.962 (0.909–1.00) |
| **NTproBNP** | 0.684 (0.575–0.792) | 0.425 (0.310–0.563) | 0.438 (0.266–0.609) | 0.864 (0.800–0.928) | 0.483 (0.301–0.665) | 0.841 (0.733–0.908) |

NTproBNP, N-terminal prohormone of brain natriuretic peptide; AUROC, area under the receiver operating characteristic curve; CI, confidence interval; AUPRC, area under the precision-recall curve

**Supplemental Table 3. Subgroup analysis**

|  | **AUROC (95% CI)** | **AUPRC (95% CI)** | **Sensitivity (95% CI)** | **Specificity (95% CI)** | **Positive predictive value (95% CI)** | **Negative predictive value (95% CI)** |
| --- | --- | --- | --- | --- | --- | --- |
| **Age** |  |  |  |  |  |  |
| < 65  (n=110) | 0.799  (0.698–0.899) | 0.661  (0.523–0.787) | 0.692  (0.515–0.870) | 0.631  (0.528–0.734) | 0.367  (0.232–0.502) | 0.869  (0.784–0.954) |
| ≥ 65  (n=131) | 0.763  (0.658–0.867) | 0.494  (0.371–0.673) | 0.714  (0.547–0.882) | 0.650  (0.558–0.743) | 0.357  (0.232–0.483) | 0.893  (0.823–0.963) |
| **Sex** |  |  |  |  |  |  |
| Male  (n=160) | 0.780  (0.695–0.864) | 0.632  (0.513–0.741) | 0.721  (0.587–0.855) | 0.658  (0.572–0.744) | 0.437  (0.321–0.552) | 0.865  (0.794–0.936) |
| Female  (n=81) | 0.778  (0.634–0.922) | 0.402  (0.223–0.666) | 0.636  (0.352–0.921) | 0.614  (0.500–0.728) | 0.206  (0.070–0.342) | 0.915  (0.835–0.995) |
| **CHA_2_DS_2_-VASc score** |  |  |  |  |  |  |
| ≥ 2 (n=177) | 0.779  (0.700–0.858) | 0.597  (0.483–0.706) | 0.733  (0.604–0.863) | 0.614  (0.531–0.697) | 0.393  (0.288–0.497) | 0.871  (0.803–0.939) |
| < 2 (n=64) | 0.749  (0.558–0.941) | 0.502  (0.268–0.755) | 0.556  (0.231–0.880) | 0.709  (0.589–0.829) | 0.238  (0.056–0.420) | 0.907  (0.820–0.994) |
| **NT-proBNP** |  |  |  |  |  |  |
| > 365 (n=167) | 0.771  (0.689–0.854) | 0.626  (0.523–0.740) | 0.689  (0.554–0.824) | 0.590  (0.503–0.677) | 0.383  (0.277–0.489) | 0.837  (0.759–0.915) |
| ≤ 365 (n=67) | 0.769  (0.564–0.974) | 0.410  (0.140–0.711) | 0.714  (0.380–1.00) | 0.767  (0.660–0.874) | 0.263  (0.065–0.461) | 0.958  (0.902–1.00) |
| **AF type** |  |  |  |  |  |  |
| Paroxysmal (n=92) | 0.773  (0.638–0.907) | 0.531  (0.367–0.729) | 0.722  (0.515–0.929) | 0.743  (0.644–0.843) | 0.406  (0.236–0.576) | 0.917  (0.847–0.987) |
| Persistent (n=149) | 0.776  (0.687–0.864) | 0.605  (0.478–0.743) | 0.694  (0.544–0.845) | 0.575  (0.484–0.666) | 0.342  (0.234–0.451) | 0.855  (0.776–0.934) |
| **AF duration** |  |  |  |  |  |  |
| > 1 year (n=78) | 0.691  (0.554–0.829) | 0.453  (0.269–0.643) | 0.529  (0.292–0.767) | 0.639  (0.519–0.760) | 0.290  (0.131–0.450) | 0.830  (0.722–0.937) |
| ≤ 1 year (n=163) | 0.818  (0.736–0.900) | 0.638  (0.518–0.757) | 0.784  (0.651–0.916) | 0.643  (0.559–0.727) | 0.392  (0.281–0.503) | 0.910  (0.851–0.970) |
| **Body mass index** |  |  |  |  |  |  |
| ≥ 25.0 (n=118) | 0.782  (0.687–0.876) | 0.521  (0.364–0.671) | 0.696  (0.508–0.884) | 0.589  (0.491–0.688) | 0.291  (0.171–0.411) | 0.889  (0.811–0.966) |
| < 25.0 (n=123) | 0.779  (0.671–0.887) | 0.672  (0.537–0.802) | 0.710  (0.550–0.869) | 0.696  (0.602–0.790) | 0.440  (0.302–0.578) | 0.877  (0.801–0.952) |
| **Heart rate** |  |  |  |  |  |  |
| ≥ 120 (n=100) | 0.785  (0.669–0.901) | 0.666  (0.514–0.805) | 0.750  (0.577–0.923) | 0.605  (0.495–0.715) | 0.375  (0.238–0.512) | 0.885  (0.798–0.971) |
| < 120 (n=141) | 0.779  (0.685–0.873) | 0.523  (0.392–0.685) | 0.667  (0.498–0.835) | 0.667  (0.579–0.754) | 0.351  (0.227–0.475) | 0.881  (0.812–0.950) |
| **Place during AF RVR** |  |  |  |  |  |  |
| Outpatient clinic (n=183) | 0.746  (0.658–0.835) | 0.508  (0.388–0.639) | 0.622  (0.465–0.778) | 0.630  (0.552–0.708) | 0.299  (0.196–0.401) | 0.868  (0.803–0.932) |
| Admission (n=16) | 0.618  (0.258–0.979) | 0.559  (0.228–0.886) | 0.600  (0.171–1.00) | 0.636  (0.352–0.921) | 0.429  (0.062–0.795) | 0.778  (0.506–1.00) |
| Emergency department (n=42) | 0.931  (0.858–1.00) | 0.833  (0.667–0.953) | 1.00  (1.00–1.00) | 0.700  (0.536–0.864) | 0.571  (0.360–0.783) | 1.00  (1.00–1.00) |
| **AF vs. atrial flutter** |  |  |  |  |  |  |
| AF (n=231) | 0.773  (0.700–0.847) | 0.571  (0.458–0.684) | 0.692  (0.567–0.818) | 0.642  (0.572–0.713) | 0.360  (0.266–0.454) | 0.878  (0.822–0.934) |
| Atrial flutter (n=10) | 0.875  (0.582–1.00) | 0.750  (0.286–1.00) | 1.00  (1.00–1.00) | 0.625  (0.290–0.960) | 0.400  (0.029–0.829) | 1.00  (1.00–1.00) |
| **Time to echocardiography** |  |  |  |  |  |  |
| within 14 days (n=144) | 0.804  (0.711–0.898) | 0.646  (0.512–0.768) | 0.781  (0.638–0.924) | 0.625  (0.535–0.715) | 0.373  (0.257–0.489) | 0.909  (0.845–0.973) |
| within 30 days (n=194) | 0.779  (0.698–0.859) | 0.585  (0.466–0.701) | 0.750  (0.622–0.878) | 0.607  (0.528–0.685) | 0.359  (0.261–0.457) | 0.892  (0.832–0.952) |
| within 60 days (n=241) | 0.777  (0.705–0.849) | 0.574  (0.466–0.677) | 0.704  (0.582–0.825) | 0.642  (0.573–0.710) | 0.362  (0.270–0.454) | 0.882  (0.828–0.937) |
| **Rhythm during echocardiography** |  |  |  |  |  |  |
| Sinus rhythm (n=60) | 0.865  (0.753–0.978) | 0.215  (0.083–0.556) | 1.00  (1.00–1.00) | 0.754  (0.643–0.866) | 0.176  (0.00–0.358) | 1.00  (1.00–1.00) |
| AF or atrial flutter (n=181) | 0.754  (0.671–0.837) | 0.632  (0.531–0.737) | 0.686  (0.559–0.814) | 0.592  (0.508–0.677) | 0.398  (0.295–0.500) | 0.828  (0.751–0.905) |
| **Left atrial diameter** |  |  |  |  |  |  |
| > 40 mm (n=183) | 0.794  (0.719–0.869) | 0.634  (0.519–0.744) | 0.750  (0.627–0.873) | 0.600  (0.517–0.683) | 0.400  (0.299–0.501) | 0.871  (0.803–0.939) |
| ≤ 40 mm (n=58) | 0.583  (0.320–0.846) | 0.288  (0.067–0.579) | 0.333  (0.00–0.711) | 0.750  (0.632–0.868) | 0.133  (0.00–0.305) | 0.907  (0.820–0.994) |

AUROC, area under the receiver operating characteristic curve; CI, confidence interval; AUPRC, area under the precision-recall curve.

NT-proBNP, N-terminal prohormone of brain natriuretic peptide; AF, atrial fibrillation; RVR, rapid ventricular response

**Supplementary Figure 1. Receiver operating characteristic curve of the secondary outcome (1-lead ECG)**

AUC is described with 95% confidence interval.

AUC, area under the receiver operating characteristic curve

**Supplementary Figure 2. Receiver operating characteristic curve within the 14-day interval**

AUC is described with 95% confidence interval.

NT-proBNP, N-terminal prohormone of brain natriuretic peptide; AUC, area under the receiver operating characteristic curve
